# Correlations between fatty acids and key aroma compounds in roasted beef cuts for flavor customization

**DOI:** 10.3389/fnut.2026.1732709

**Published:** 2026-02-02

**Authors:** Ningbo Wang, Yingying Zhong, Haiqiang Zhu, Haiying Zhao

**Affiliations:** 1 School of Food Science and Biotechnology, Zhejiang Gongshang University, Hangzhou, China; 2 Ningbo Customs Technology Center, Ningbo, China; 3 Ningbo Joysun Product Testing Service Company, Ningbo, China

**Keywords:** biochemical pathways, correlation network, fatty acidomics, flavor customization, HS-SPME-GC-TOFMS, lipid oxidation

## Abstract

Lipids are crucial determinants of the flavor and nutritional quality of meat. However, a deep understanding of how specific fatty acids direct the formation of key aroma compounds during thermal processing remains a challenge. This study employed an innovative fatty acidomics approach combined with HS-SPME-GC-TOFMS to systematically investigate the relationship between the lipid composition of six beef cuts (with three biological replicates per cut) and the volatile aroma profiles generated upon roasting. Multivariate statistics and correlation network analysis revealed that ultra-long-chain saturated fatty acids (C21:0, C22:0) showed strong positive correlations with fruity and cheesy aroma-related ketones (2-octanone, 2-heptanone), while the monounsaturated fatty acid C18:1n9c was significantly correlated with mushroom-alcohol (1-octen-3-ol). These flavors were formed through thermal degradation of saturated fatty acids and the specific 10-hydroperoxide cleavage of oleic acid. Conversely, polyunsaturated fatty acids such as C20:3n3 and C18:2n6t exhibited a significant negative correlation with dimethyl trisulfide, an undesirable sulfurous off-flavor compound. This suggests a competitive inhibition mechanism whereby rapid PUFA oxidation consumes reactive intermediates, thereby suppressing the Maillard reaction pathway responsible for off-flavor formation. Our findings provide novel biochemical insights into how the lipid matrix directly generates positive flavors and indirectly shapes the overall aroma profile. This work provides a theoretical basis for the targeted customization of beef flavor through precise regulation of lipid composition, aligning with the growing demand for nutrition-oriented and sensorially optimized foods.

## 1 Introduction

Flavor is a critical factor determining the quality and consumer acceptance of meat products. The distinctive aroma of roast meat primarily arises from the synergistic effects of complex chemical pathways, including lipid oxidation, the Maillard reaction, and thiamine degradation (1). Among these, lipid oxidation is considered the core pathway for forming volatile flavor compounds, contributing approximately 90% of the aromatic substances in cooked meat (2). During heating, unsaturated fatty acids in meat undergo oxidative cleavage to generate low-threshold volatile compounds like aldehydes, ketones, and alcohols, imparting the characteristic aroma of roast meat (3). The natural variation in intermuscular fat content and composition in different parts of beef combines high consumer preference with economic value, making beef an ideal model for studying lipid-driven flavor formation (4).

Mechanistic studies using model systems have shown that each UFA produces a unique volatile fingerprint. Oleic acid (C18:1 n-9) preferentially forms 2-decenal and 1-octen-3-ol (4), linoleic acid (C18:2 n-6) gives pentanal, hexanal and 2-heptanone via both 9- and 13-hydroperoxide intermediates, and α-linolenic acid (C18:3 n-3) generates 2,4-heptadienal and 3,6-nonadienal (5, 6). At roasting temperatures (180 °C−250 °C) these hydroperoxides are short-lived and decompose homolytically, the resulting alkyl and alkoxyl radicals undergo β-scission to release C5–C10 volatiles that survive long enough to be sensed (7). Saturated fatty acid content (SFAs), lacking double bonds, oxidize at the β-scission of alkoxyl radicals, releasing C3–C5 methyl ketones and γ-lactones (8). Consequently, the aldehydes, ketones, and lactone volatile chemicals of beef roast flavor are determined by the relative content of SFAs, Monounsaturated fatty acid content (MUFAs), and polyunsaturated fatty acids (PUFA) (9).

Although the chemistry outlined above is well established in heated triolein or trilinolein models (10), its quantitative translation to real beef cuts remains fragmentary. Most studies have compared breeds or aging regimes, but systematic investigations linking the intrinsic fatty-acid profile of individual retail cuts to their grilled volatile spectra are scarce (11, 12).

This work aims to identify site-specific precursors for flavor tailoring in roasted beef cuts. Six commercially high-value beef cuts (Chuck Roll (CR), Short Ribs (SR), Skirt Steak (SS), Top Round (TR), Ribeye Cap (RC), Oyster Blade (OB)) with varied lipid profiles were studied. The fatty acid profiles in raw beef cuts and key aroma compounds upon roasting were identified and investigated using headspace solid-phase microextraction-gas chromatography-time-of-flight mass spectrometry (HS-SPME-GC-TOFMS). Mapping of fatty acids and volatiles was conducted by multivariate statistics and correlation network analysis. Our findings can provide a chemical basis for predicting and controlling roast-beef aroma based on raw-beef fatty acid profiles.

## 2 Materials and methods

### 2.1 Beef sample preparation and roast processing

Frozen Chuck Roll (CR), Short Ribs (SR), Skirt Steak (SS), Top Round (TR), Ribeye Cap (RC), and Oyster Blade (OB) beef tissues were purchased from local commercial suppliers. Each cut was homogenized and formed into 1 cm thick, 5 cm diameter patties. Samples were roasted in an oven at 200 °C for 10 min, as per previously optimized conditions (13). The roasted beef tissue samples were cooled at room temperature and finely chopped for subsequent fatty acid and volatile compound analysis. The moisture content was determined using the direct drying method stipulated in the Chinese National Food Safety Standard GB 5009.3-2016. The fat content was determined following the methods acid hydrolysis described in GB 5009.6-2025. The six beef tissues and moisture and fat content were listed in Supplementary Table S1.

### 2.2 Chemicals and materials

Chromatography grade acetone and hexane were purchased from Merck (Germany). The required internal standard 1,2-dichlorobenzene, CAS No.:95-50-1, was purchased from Alta Science Co., Ltd (Tianjin, China, First Standard^®^). The fatty acid standards were 37 fatty acid methyl esters mixed standard CRM47885 from SUPELCO (Sigma, USA).

### 2.3 Chromatographic analysis of fatty acids

The gas chromatographic analysis of fatty acids was referred to the research method of Qiao et al. in which the pre-treatment of fatty acid methylation was performed by alkali-catalyzed method (14). Weigh 1 g of beef tissue sample, put it in a 15 ml centrifuge tube, add 5 ml chloroform-methanol (1:1, V/V), 1 ml of ultrapure water, and extract it by shaking for 10 min, centrifuge to make the aqueous and organic phases stratified. The lower organic layer was aspirated into a heart-shaped flask, and then 5 ml of chloroform was added to the centrifuge tube, and the extraction was repeated once. The extracts were combined and rotary evaporated to near dryness. Add 2 ml of 2 M potassium hydroxide in methanol to the extract, vortexing and shaking for 1 min; add 2 ml of distilled water, 5 ml of n-hexane, shaking for 1 min; then add an appropriate amount of anhydrous sodium sulfate, centrifugation, the supernatant was collected and filtered through a 0.45 μm organic phase microporous membrane, and then collect the filtrate in the chromatography injection vial for the subsequent gas chromatographic analysis.

The 37 fatty acid standards and samples were analyzed by chromatography on an Agilent 7890B GC system with a SP-2560 column (SUPELCO, Sigma, USA) of 100 m length, 0.25 mm inner diameter, and 0.2 μm film thickness, and the injector temperature was 270 °C. Detector temperature: 280 °C. Program temperature: initial temperature 100 °C, 13 min; 100 °C−180 °C, temperature increase rate 10 °C/min, keep 6 min; 180 °C−200 °C, temperature increase rate 1 °C/min, keep 20 min; 200 °C−230 °C, temperature increase rate 4 °C/min, keep 10.5 min. carrier gas: nitrogen. Split ratio: 100:1, injection volume: 1.0 μl. The calibration curves, recoveries and detection limits of the 37 fatty acid standards were shown in Supplementary Table S2, and the chromatograms of the 37 fatty acid standards were shown in Supplementary Figure S1.

### 2.4 Headspace solid-phase microextraction (HS-SPME) for extraction of volatile compounds

Volatile compounds were extracted using the HS-SPME method described by Jiang et al. with modifications (15). Briefly, refrigerated beef samples were ground into a paste in a blender. Precise weights of 4 g each of the six prepared beef samples were placed into 20 ml headspace vials. Next, 5 ml of saturated sodium chloride solution (NaCl, Sinopharm Group, Shanghai) was added, along with 1 μl of 1,2-dichlorobenzene (0.1 mg/ml methanol solution) as an internal standard for semiquantitative analysis of Volatile organic compounds (VOCs). The determined concentration values were suitable for comparing the relative abundance between different beef samples, but the differences in SPME adsorption efficiency of different compounds were not considered. After preheating the headspace vial in a 60 °C water bath for 20 min, the SPME needle was inserted into the vial. Extend the fiber tip (DVB/CAR/PDMS, 50/30 μm, Supelco, USA) and maintain 30 min to extract volatile compounds. Subsequently, the SPME needle was rapidly inserted into the gas chromatography (GC) inlet. When the fiber was extended and held at 270 °C for 5 min, the volatile compounds underwent complete thermal desorption.

### 2.5 Determination of volatile compounds

To separate volatile compounds in the sample, detection was performed using a gas chromatography-time-of-flight mass spectrometer (GC-TOFMS) equipped with an Rtx-5MS capillary column (30 m × 0.25 mm × 0.25 μm, Supelco, USA), with the GC inlet temperature set to 270 °C. For GC-TOFMS analysis, the programmed temperature ramp described in was established with modifications. The initial column temperature was maintained at 40 °C for 3 min, then steadily increased at 5 °C/min to 150 °C, followed by a steady rise at 15 °C/min to 240 °C, where it was held for 5 min. High-purity helium was used as the carrier gas at a flow rate of 1 ml/min. The injection port operated in splitless mode with a solvent delay of 1 min. Additionally, mass spectrometry parameters were established: the ion source electron energy was set to 70 eV. The MS interface temperature was set to 280 °C, and the ion source temperature was adjusted to 230 °C. The scan range was adjusted to 30–500 m/z.

All volatile compounds were identified using the National Institute of Standards and Technology (NIST 20) mass spectrometry library and compared with the corresponding retention indices (RI) reported in previous literature. Peak retention times of unknown compounds and n-alkane mixtures (C7-C40, purchased from ANPEL-TRACE Standard Technical Service Co., Ltd., Shanghai, China) were compared under identical conditions in GC-TOFMS. The content of volatile compounds was calculated using 1,2-dichlorobenzene (0.1 mg/ml methanol solution) as an internal standard.

### 2.6 Calculation of odor activity value

To identify individual volatile compounds influencing the overall flavor of meat, the concept of Odor Activity Value (OAV) was introduced. It serves as a key indicator for determining the contribution intensity of a single volatile compound to the overall flavor of a sample. OAV depends not only on the concentration of the volatile compound but also on its odor threshold. OAV is calculated by dividing the volatile compound concentration by its odor threshold in water. An OAV ≥ 1 indicates that the compound contributes significantly to the food's overall flavor, with higher values indicating a greater contribution. Conversely, an OAV < 1 suggests a minor direct contribution (16).

### 2.7 Statistical analysis

Orthogonal Partial Least Squares Discriminant Analysis (OPLS-DA), Biplot analysis and Variable Importance Analysis (VIP) were performed on the aroma compounds of Roast Beef using SIMCA software (version 14.1, Umetrics, Umea, Sweden). The compounds with VIP ≥ 1.0 indicated a significant difference. Correlation network analysis of VOCs and fatty acids was performed using pearson correlation algorithm with *P* < 0.01 and correlation values rho > 0.5 (positive), rho < −0.5 (negative) (https://www.omicstudio.cn/tool). Calculated values of correlation coefficients are displayed in the Supplementary material Pearson_Corr_Data.xlsx. To ensure data robustness, all experimental samples were measured in triplicate. Data are presented as mean ± standard deviation (Mean ± SD). Different lowercase letters indicate statistically significant differences among cuts as determined by one-way analysis of variance (ANOVA) followed by Tukey's HSD test (*P* < 0.05). Analysis results were visualized using GraphPad 8.0.2 software.

## 3 Results

### 3.1 Characteristics of fatty acid composition in different beef tissues

**Figure 1:**
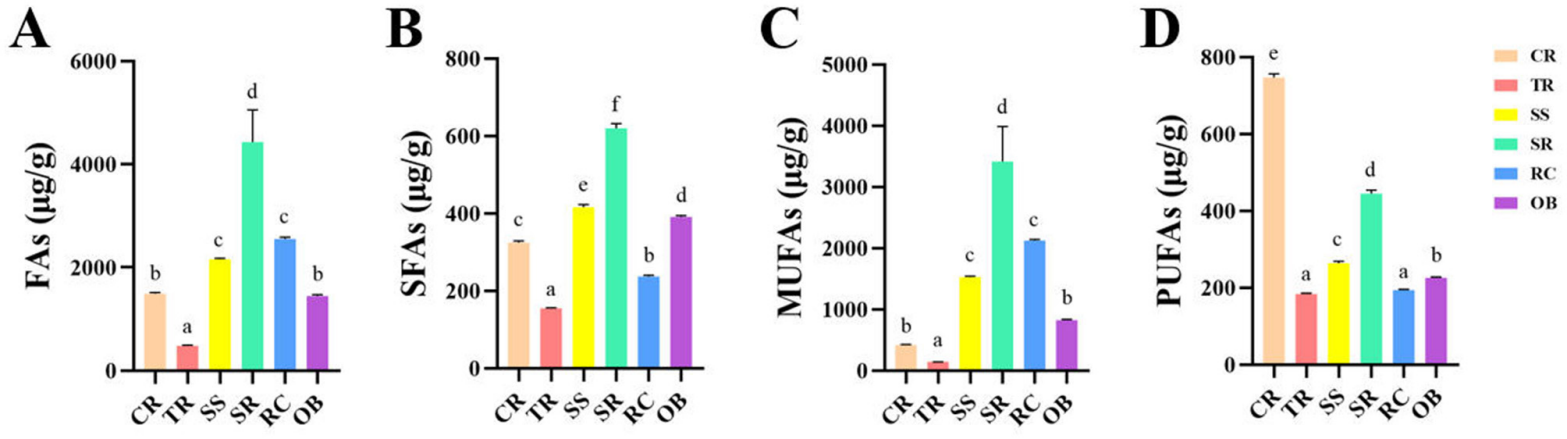
Total fatty acid content (FAs) **(A)**, saturated fatty acid content (SFAs) **(B)**, monounsaturated fatty acid content (MUFAs) **(C)**, polyunsaturated fatty acid content (PUFAs) **(D)** in roast beef. Data are presented as mean ± standard deviation (*n* = 3). Different lowercase letters indicate statistically significant differences among cuts as determined by one-way ANOVA followed by Tukey's HSD test (*P* < 0.05).

The fatty acid composition of six distinct beef cuts (CR, TR, SS, SR, RC, OB) is presented in Table 1. As shown in Figure 1, significant differences (*P* < 0.05) were observed among cuts in total fatty acid content (FAs), SFA and UFA composition, and their respective proportions. Detailed results of the one-way ANOVA for pairwise comparisons of fatty acid content among beef cuts and Tukey's HSD *post hoc* test are presented in Supplementary Table S3. Total fatty acid content decreased in the following order: SR (4,434.28 μg/g) > RC (2,553.54 μg/g) > SS (2,155.10 μg/g) > CR (1,490.18 μg/g) > OB (1,449.28 μg/g) > TR (484.22 μg/g). The SR region exhibited the highest total fatty acid content (4,434.28 μg/g), approximately nine times that of the lowest region, TR (484.22 μg/g). SFA proportion was highest in TR (32.73%) and lowest in RC (10.84%). Among specific SFA types, C14:0 content was significantly higher in CR (205.25 μg/g), while C15:0 was relatively higher in SS, SR, RC, and OB, the C23:0 was distributed across all tissues. UFAs constituted the primary fatty acid fraction in roast beef, exceeding 65% across all cuts. The RC had the highest UFA proportion (89.16%), while the TR had the lowest (67.27%). Among MUFAs, C18:1n9c was particularly prominent in the SR and RC, with an average concentration of 2,599.69 μg/g in the SR. Among PUFAs, C18:2n6c was highest in CR and lowest in OB. C18:3n3 was present in multiple cuts but generally lower than C18:2n6c. Overall, SR and RC not only exhibited high total fatty acid content but also significantly higher levels and proportions of C18:1n9c compared to other cuts, while TR and OB showed a higher proportion of saturated fatty acids.

**Table 1:** Fatty acid content (μg/g) in roast beef (means ± SD, *n* = 3).

**Fatty acids**	**CR**	**TR**	**SS**	**SR**	**RC**	**OB**
C10:0	0 ± 0 a	0 ± 0 a	13.84 ± 0.4 c	13.85 ± 0.43 c	7.61 ± 0.2 b	12.21 ± 0.17 c
C12:0	6.41 ± 0.11 b	0 ± 0 a	21.63 ± 0.49 d	27.95 ± 0.86 e	13.19 ± 0.31 c	19.85 ± 0.32 d
C13:0	0 ± 0 a	0 ± 0 a	5.52 ± 0.15 b	7.37 ± 0.24 c	0 ± 0 a	5.48 ± 0.08 b
C14:0	205.25 ± 2.98 c	77.56 ± 0.97 b	0 ± 0 a	0 ± 0 a	0 ± 0 a	0 ± 0 a
C14:1n5	39.91 ± 0.59 b	13.67 ± 0.23 a	125.25 ± 3.05 d	259.52 ± 7.59 e	92.87 ± 1.39 c	307.43 ± 4.97 f
C15:0	27.71 ± 0.39 b	16.56 ± 0.27 a	147.4 ± 3.45 d	214.92 ± 7.19 e	96.94 ± 1.49 c	152.68 ± 2.09 d
C15:1n5	0 ± 0 a	0 ± 0 a	0 ± 0 a	6.61 ± 0.2 b	0 ± 0 a	6.34 ± 0.15 b
C16:1n7	33.29 ± 0.52 b	22.47 ± 0.22 a	58.76 ± 1.32 c	61.96 ± 0.42 d	34.7 ± 0.3 b	56.6 ± 1.29 c
C17:0	8.58 ± 0.1 a	36.78 ± 0.63 d	27.16 ± 0.61 c	63.35 ± 1.45 e	18.3 ± 0.21 b	78.66 ± 1.49 f
C17:1n7	41.13 ± 0.64 b	20.53 ± 0.26 a	236.02 ± 5.69 d	414.72 ± 10.66 f	117.32 ± 1.71 c	356.8 ± 6.19 e
C18:1n9c	297.84 ± 3.86 c	85.68 ± 1.09 b	969.49 ± 8.51 d	2600.36 ± 569.88 f	1830.11 ± 23.39 e	0 ± 0 a
C18:2n6t	6.47 ± 0.07 b	0 ± 0 a	39.45 ± 0.99 c	49.04 ± 1.19 d	57.93 ± 0.9 e	36.69 ± 0.87 c
C18:2n6c	482.81 ± 6.06 f	83.32 ± 0.87 d	59.44 ± 0.99 c	116.92 ± 3.62 e	25.63 ± 0.5 b	19.85 ± 0.12 a
C20:0	8.28 ± 0.08 c	0 ± 0 a	16.25 ± 1.75 d	3.56 ± 0.07 b	7.6 ± 0.11 c	38.9 ± 0.79 e
C18:3n3	232.96 ± 3.37 d	94.13 ± 1.24 a	147.4 ± 3.45 b	214.92 ± 7.19 c	96.94 ± 1.49 a	152.68 ± 2.09 b
C20:1	5.64 ± 0.15 b	0 ± 0 a	34.69 ± 0.92 d	35.3 ± 0.87 d	29.87 ± 0.39 c	40 ± 0.73 e
C21:0	21.89 ± 0.33 c	8.79 ± 0.16 b	53.41 ± 1.44 e	208.11 ± 6.48 f	36.23 ± 0.89 d	7.25 ± 0.17 a
C20:2	0 ± 0 a	0 ± 0 a	10.82 ± 0.37 d	10.19 ± 4 d	7.72 ± 0.18 c	5.27 ± 0.11 b
C22:0	0 ± 0 a	0 ± 0 a	5.18 ± 0.34 b	14.89 ± 0.49 d	6.72 ± 0.27 c	0 ± 0 a
C20:3n6	14.9 ± 0.2 c	5.94 ± 0.09 b	0 ± 0 a	0 ± 0 a	0 ± 0 a	0 ± 0 a
C22:1n9	0 ± 0 a	0 ± 0 a	48.62 ± 1.37 e	35.09 ± 1.11 c	14.65 ± 0.25 b	44.73 ± 0.76 d
C20:3n3	10.54 ± 0.16 c	0 ± 0 a	9.81 ± 0.28 c	6.98 ± 0.26 b	6.63 ± 0.14 b	11.15 ± 0.21 d
C23:0	46.59 ± 0.68 b	18.78 ± 0.27 a	124.98 ± 3.54 f	68.66 ± 2.42 d	52.58 ± 0.87 c	96.73 ± 1.91 e

Significant differences (*P* < 0.05; one-way ANOVA, Tukey's HSD test) among cuts within indicated by different lowercase letters.

**Figure 2:**
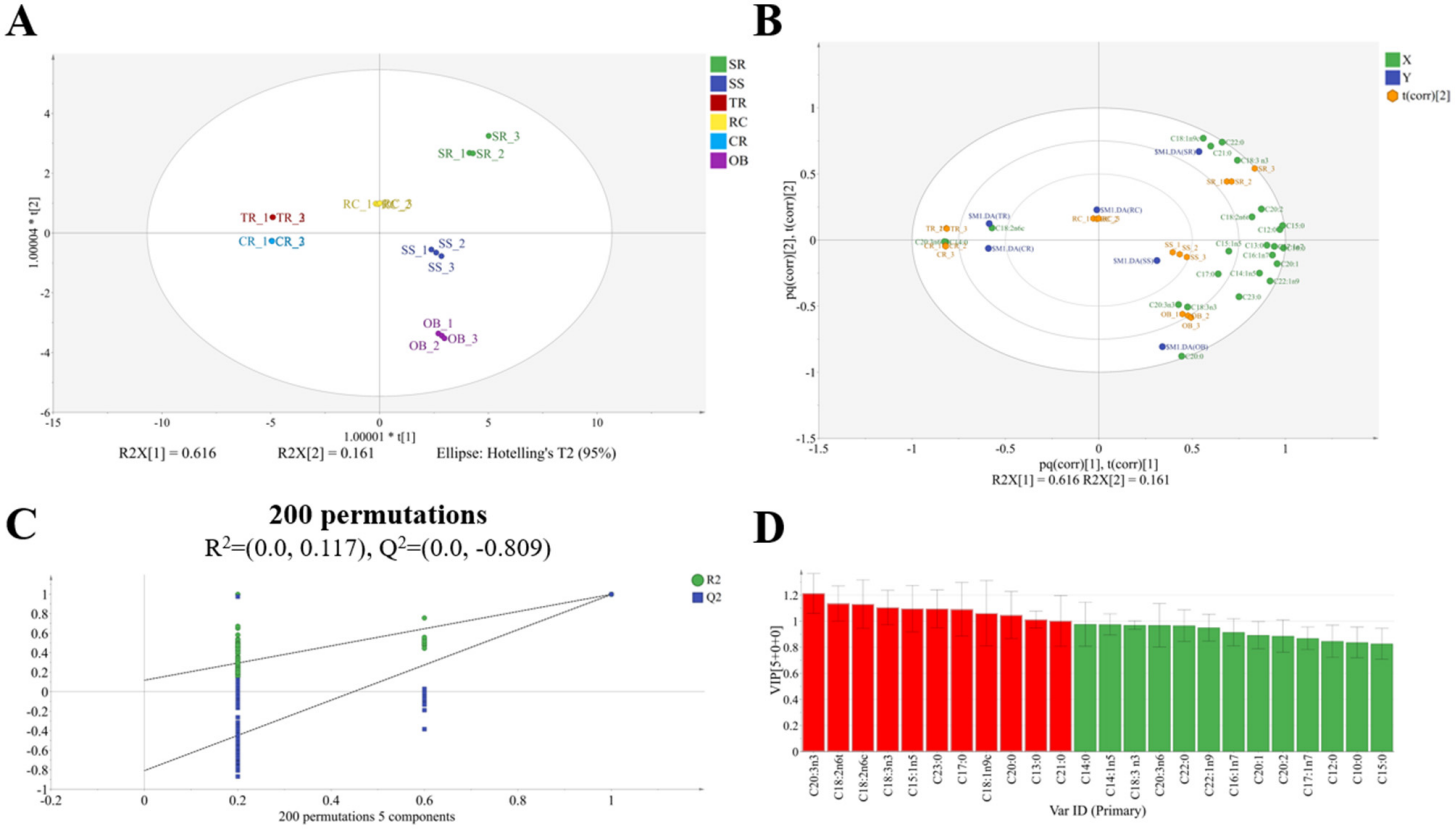
OPLS-DA analysis of fatty acids in roast beef **(A)**, biplot analysis **(B)**, permutation score plots **(C)**, and VIP fatty acids (VIP > 1) **(D)**.

OPLS-DA completely distinguished the fatty acid profiles of the six cuts (Figure 2A, *R*^2^*X* = 0.996, *R*^2^*Y* = 0.997, *Q*^2^ = 0.995). Biplot analysis further indicated that C18:2n6c, C20:3n6, and C14:0 were characteristic fatty acids for CR and TR; C20:3n3, C18:3n3, and C20:0 were characteristic fatty acids for OB; while C20:3n3, C18:1n9c, C21:0, and C22:0 constituted the characteristic fatty acid profile for SR; the fatty acid compositions of RC and SS were relatively balanced (Figure 2B). 200 permutations validated the model's good reliability (Figure 2C). As shown in Figure 2D, Supplementary Table S4 and Supplementary Figure S2, VIP analysis further identified C18:1n9c as the major fatty acid responsible for differences in SS, SR, and RC, while C18:2n6c was predominant in CR.

### 3.2 Analysis of key aromatic compounds in roast beef

**Figure 3:**
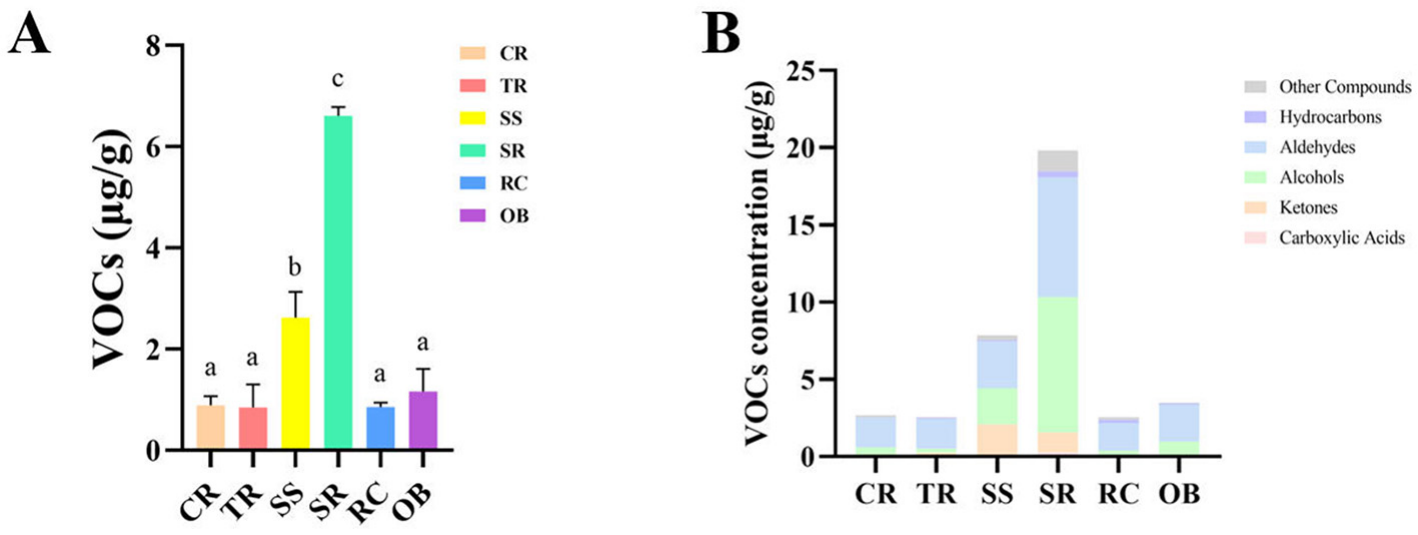
VOCs content **(A)** and VOCs classification **(B)** in roast beef. Data are presented as mean ± standard deviation (*n* = 3). Different lowercase letters indicate statistically significant differences among cuts as determined by one-way ANOVA followed by Tukey's HSD test (*P* < 0.05).

VOCs were analyzed in six distinct beef cuts (CR, TR, SS, SR, RC, OB), identifying a total of 30 volatile compounds. These encompassed aldehydes, ketones, alcohols, hydrocarbons, heterocyclic compounds, and sulfur-containing compounds, as listed in Table 2. As shown in Figure 3, significant differences (*P* < 0.05) (Supplementary Table S5) in VOC composition existed among the cuts, reflecting tissue-specific flavor precursors. Total VOC content was significantly higher in SS and SR (*P* < 0.05) at 2.625 μg/g and 6.609 μg/g, respectively. Aldehydes and alcohols constitute the primary aroma components of roast beef (Figure 3B). SR exhibited the highest levels of both aldehydes (7.76 μg/kg) and alcohols (8.77 μg/kg), followed by OB. In contrast, TR was dominated by aldehydes (1.93 μg/kg) and ketones (0.25 μg/kg).

**Table 2:** Volatile compounds concentration in roast beef (μg/kg) (means ± SD, *n* = 3).

**Classification**	**Volatile compounds**	**CR**	**TR**	**SS**	**SR**	**RC**	**OB**
Carboxylic acids	Hexanoic acid	0 ± 0 a	0 ± 0 a	0 ± 0 a	91.19 ± 79.51 b	0 ± 0 a	0 ± 0 a
Ketones	2,3-Octanedione	0 ± 0 a	56.02 ± 97.03 b	630.81 ± 970.25 c	250.53 ± 48.3 c	17.75 ± 16.5 b	25.89 ± 23.06 b
	2-Heptanone	22.86 ± 5.27 ab	17.19 ± 9.71 a	54.83 ± 30.5 b	103.97 ± 14.9 c	9.57 ± 3.1 a	15.63 ± 4.3 a
	2-Octanone	2.13 ± 3.69 b	0 ± 0 a	6.17 ± 10.68 b	69.36 ± 9.59 c	0 ± 0 a	0 ± 0 a
	2-Butanone, 3,4-epoxy-3-ethyl-	0 ± 0 a	8.64 ± 8.85 b	0 ± 0 a	0 ± 0 a	8.53 ± 14.77 b	0 ± 0 a
Alcohols	1-Hexanol	27.12 ± 5.6 b	0 ± 0 a	28.09 ± 4.35 b	165.01 ± 31.98 c	17.95 ± 3.94 ab	10.35 ± 17.92 a
	1-Octen-3-ol	146.24 ± 85.01 a	89.34 ± 121.21 a	744.36 ± 82.7 b	2688.44 ± 468.96 c	77.5 ± 62.84 a	270.43 ± 264.27 a
	2-Octen-1-ol, (Z)-	0 ± 0 a	0 ± 0 a	11.09 ± 19.2 a	70.31 ± 46.37 b	0 ± 0 a	0 ± 0 a
Aldehydes	2-Heptenal, (Z)-	0 ± 0 a	0 ± 0 a	4.17 ± 7.22 a	34.64 ± 34.06 a	0 ± 0 a	0 ± 0 a
	2-Nonenal, (E)-	0 ± 0 a	0 ± 0 a	3.11 ± 5.38 a	30.94 ± 31.63 a	0 ± 0 a	0 ± 0 a
	2-Octenal, (E)-	0 ± 0 a	0 ± 0 a	13.21 ± 11.59 a	25.06 ± 43.41 a	0 ± 0 a	0 ± 0 a
	Benzaldehyde	122.64 ± 10.15 a	289.47 ± 211.23 ab	122.95 ± 36.28 a	218.55 ± 40.35 b	213.99 ± 60.28 ab	351.05 ± 52.3 b
	Decanal	0 ± 0 a	0 ± 0 a	0 ± 0 a	9.69 ± 16.79 a	0 ± 0 a	0 ± 0 a
	Heptanal	35.49 ± 18.2 a	24.57 ± 12.67 a	98.45 ± 37.49 b	743.53 ± 292.07 c	26.91 ± 20.8 a	83.34 ± 42.64 a
	Hexanal	357.46 ± 145.06 b	192.91 ± 28.84 a	456.91 ± 392.49 ab	1023.63 ± 872.57 b	313.82 ± 153.08 a	106.12 ± 119.16 a
	Nonanal	110.67 ± 55.94 b	111.58 ± 84.95 b	219.55 ± 18.97 b	252.19 ± 417.71 ab	19.48 ± 16.16 a	178.29 ± 92.95 b
	Octanal	16.75 ± 18.09 a	26.08 ± 10.23 a	95.64 ± 26.91 b	246.95 ± 94.41 c	19.59 ± 6.5 a	80.16 ± 70.13 a
Hydrocarbons	Decane	0 ± 0 a	0 ± 0 a	0 ± 0 a	0 ± 0 a	3.62 ± 4.94 a	0 ± 0 a
	Dodecane	0 ± 0 a	9.22 ± 4.19 b	11.31 ± 5 b	27.29 ± 7.47 c	12.69 ± 1.11 b	10.5 ± 9.06 ab
	Hexadecane	0 ± 0 a	1.21 ± 2.1 a	0 ± 0 a	0 ± 0 a	10.51 ± 6.36 b	0.03 ± 0.06 a
	Tetradecane	0 ± 0 a	4.35 ± 1.57 b	0 ± 0 a	7.37 ± 12.76 ab	0.9 ± 1.55 a	2.95 ± 2.66 ab
	Tridecane	0 ± 0 a	1.07 ± 1.86 a	9.57 ± 3.69 b	12.32 ± 10.67 b	0 ± 0 a	0 ± 0 a
	Undecane	0 ± 0 a	0.88 ± 1.53 a	0 ± 0 a	69.3 ± 15.04 c	21.62 ± 15.77 b	11.38 ± 10.46 a
	Tridecane, 3-methyl-	0 ± 0 a	2.27 ± 2.26 a	0 ± 0 a	0 ± 0 a	0 ± 0 a	0.36 ± 0.63 a
	Undecane, 3-methyl-	0 ± 0 a	0 ± 0 a	0 ± 0 a	0 ± 0 a	5.28 ± 3.07 b	0 ± 0 a
	1,3,5,7-Cyclooctatetraene	2.05 ± 3.56 a	0 ± 0 a	4.58 ± 7.94 a	16.47 ± 14.4 ab	12.13 ± 2.48 b	0 ± 0 a
Nitrile	4-Cyanocyclohexene	20.51 ± 6.57 b	0 ± 0 a	0 ± 0 a	90.81 ± 57.3 c	0 ± 0 a	0 ± 0 a
Sulfide	Dimethyl trisulfide	0 ± 0 a	1.72 ± 0.44 b	0 ± 0 a	0 ± 0 a	0 ± 0 a	0 ± 0 a
Heterocyclic	Furan, 2-pentyl-	0 ± 0 a	0 ± 0 a	71.96 ± 124.65 a	259.62 ± 282.98 a	47.69 ± 43.75 a	0 ± 0 a
Oxime	Oxime-, methoxy-phenyl-_	28.73 ± 5.7 b	12.29 ± 7.92 a	37.8 ± 24.86 ab	101.6 ± 6.46	13.79 ± 9 a	14.71 ± 13.24 a

Significant differences (*P* < 0.05; one-way ANOVA, Tukey's HSD test) among cuts within indicated by different lowercase letters.

Regarding specific compounds, the OAV values of characteristic VOCs serve as a key basis for evaluating odor impact. Table 3 lists the OAV and aroma types of major aroma compounds. Among these, hexanal exhibited the highest concentration in SR (1.02 μg/kg). It is widely recognized as a key odorant contributing green and fresh notes in meat aroma and serves as a classic marker for the oxidation of linoleic acid, which was notably abundant in the SR cut (17). Heptanal is also prominent in SR, imparting fatty and fruity notes. Benzaldehyde is most abundant in OB, contributing a bitter almond aroma. Nonanal and octanal are widely present, collectively forming fatty and citrus-like aromas.

**Table 3:** The odor descriptions and OAV of the aroma compounds in roast beef (means ± SD, n = 3).

**Compound name**	**CAS number**	**Odor descriptions**	**Threshold (μg/kg)**	**OAV**					
				**CR**	**TR**	**SS**	**SR**	**RC**	**OB**
2,3-Octanedione	585-25-1	Herbal	3	0 ± 0	0.02 ± 0.03	0.21 ± 0.32	0.08 ± 0.02	0.01 ± 0.01	0.01 ± 0.01
2-Heptanone	110-43-0	Cheesy, fruity	0.14	0.16 ± 0.04	0.12 ± 0.07	0.39 ± 0.22	0.74 ± 0.11	0.07 ± 0.02	0.11 ± 0.03
2-Octanone	111-13-7	Cheesy, fruity	0.0502	0.04 ± 0.07	0 ± 0	0.12 ± 0.21	1.38 ± 0.19	0 ± 0	0 ± 0
1-Octen-3-ol	3391-86-4	Mushroom	0.0015	97.49 ± 56.67	59.56 ± 80.8	496.24 ± 55.14	1792.29 ± 312.64	51.67 ± 41.89	180.29 ± 176.18
Benzaldehyde	100-52-7	Fruity	0.75089	0.16 ± 0.01	0.39 ± 0.28	0.16 ± 0.05	0.29 ± 0.05	0.28 ± 0.08	0.47 ± 0.07
Heptanal	111-71-7	Fresh, green	0.0028	12.67 ± 6.5	8.77 ± 4.52	35.16 ± 13.39	265.55 ± 104.31	9.61 ± 7.43	29.76 ± 15.23
Hexanal	66-25-1	Fresh, green	0.005	71.49 ± 29.01	38.58 ± 5.77	91.38 ± 78.5	204.73 ± 174.51	62.76 ± 30.62	21.22 ± 23.83
Nonanal	124-19-6	Aldehydic, citrus	0.0011	100.61 ± 50.86	101.43 ± 77.23	199.59 ± 17.25	229.27 ± 379.73	17.7 ± 14.69	162.08 ± 84.5
Octanal	124-13-0	Aldehydic, green	0.0008	20.94 ± 22.61	32.6 ± 12.79	119.55 ± 33.64	308.68 ± 118.01	24.49 ± 8.13	100.2 ± 87.66
Dimethyl trisulfide	3658-80-8	Sulfurous	0.0001	0 ± 0	17.16 ± 4.4	0 ± 0	0 ± 0	0 ± 0	0 ± 0
2-pentylfuran	3777-69-3	Fruity, green	0.0058	0 ± 0	0 ± 0	12.41 ± 21.49	44.76 ± 48.79	8.22 ± 7.54	0 ± 0

Odor descriptions obtained from the Perflavory Information System (http://www.perflavory.com/).

Among alcohols, 1-octen-3-ol reached 2.69 μg/kg in SR, exhibiting a characteristic mushroom flavor closely linked to UFA oxidation. For ketones, 2,3-octanedione was highest in SS (0.63 μg/kg), contributing mushroom and creamy aromas. while 2-heptanone and 2-octanone are relatively abundant in SR, contributing fruity and cheesy flavors respectively. Hydrocarbon compounds are present at lower levels, making limited contributions to the overall flavor profile. 2-Pentylfuran is relatively abundant in both SR and SS, imparting a bean-like and grassy odor, while dimethyl trisulfide detected in TR contributes a sulfur-like odor.

**Figure 4:**
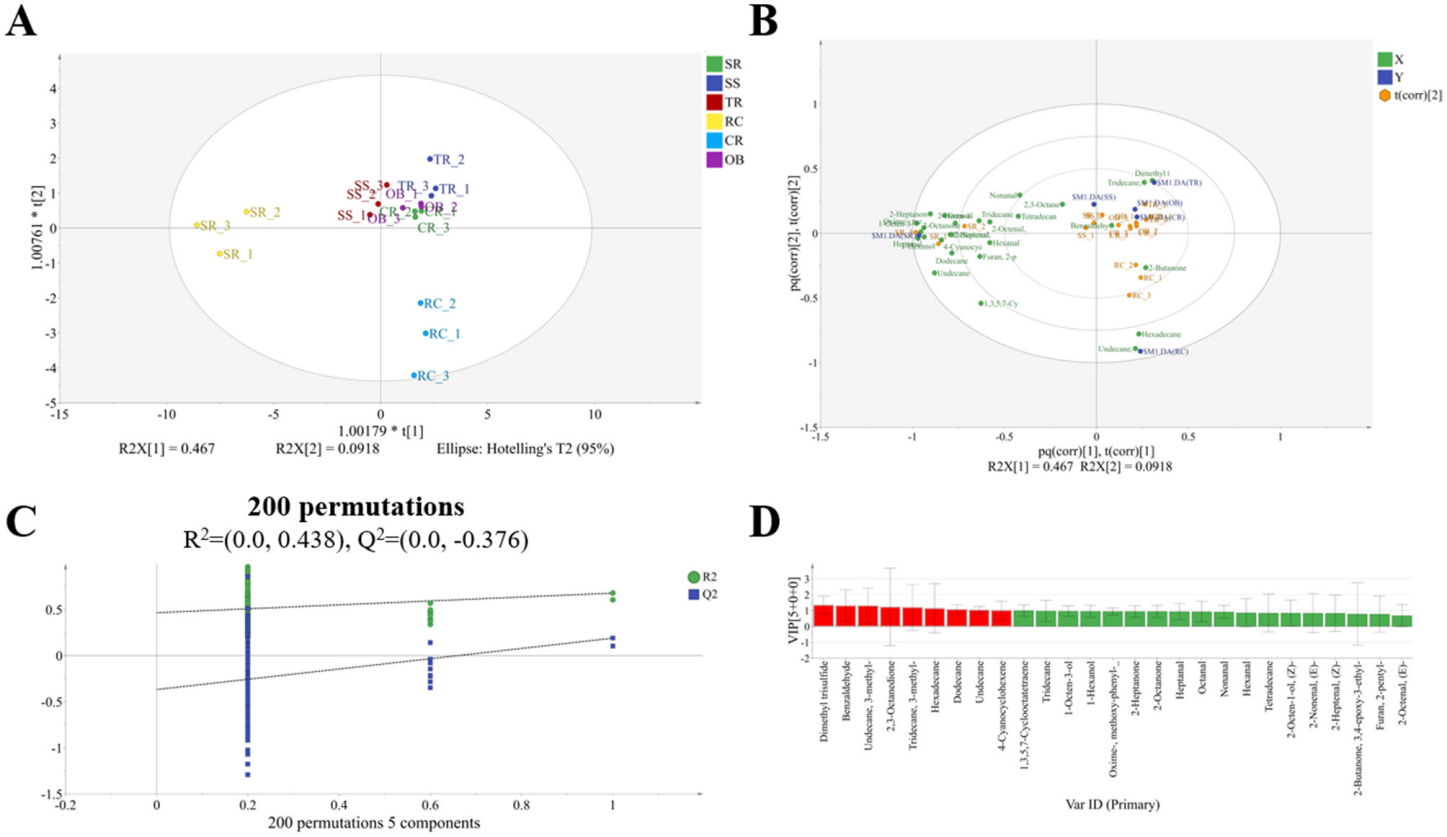
OPLS-DA analysis of VOCs in roast beef **(A)**, biplot analysis **(B)**, permutation score plots **(C)**, and VIP volatile compounds (VIP > 1) **(D)**.

As shown in Figure 4, OPLS-DA analysis of roast beef VOCs indicates that the volatile profiles of RC and SR are distinctly separated from other cuts, while the VOCs of TR, OB, and CR tend to overlap. The OPLS-DA model established for analyzing VOCs yielded parameters *R*^2^*X, R*^2^*Y*, and *Q*^2^ of 0.85, 0.868, and 0.597, respectively. Biplot analysis revealed that numerous alcohol, aldehyde, and ketone VOCs including 1-Octen-3-ol and 2-Heptanone emerged as signature VOCs for SR tissue. Dimethyl trisulfide was the signature VOC for TR, while Hexadecane, Undecane, and 3-Methyl- became signature VOCs for RC tissue. A 200-permutation test validated the classification model's reliability (Figure 4C). Further analysis identified nine VIP > 1 VOCs (Supplementary Table S6): dimethyl trisulfide, benzaldehyde, undecane, 3-methyl-, 2,3-octanedione, tridecane, 3-methyl-, hexadecane, dodecane, undecane, and 4-cyanocyclohexene. These compounds are key VOCs constituting the aroma of roasted beef from different cuts.

### 3.3 Correlation network analysis between fatty acids and aroma compounds

**Figure 5:**
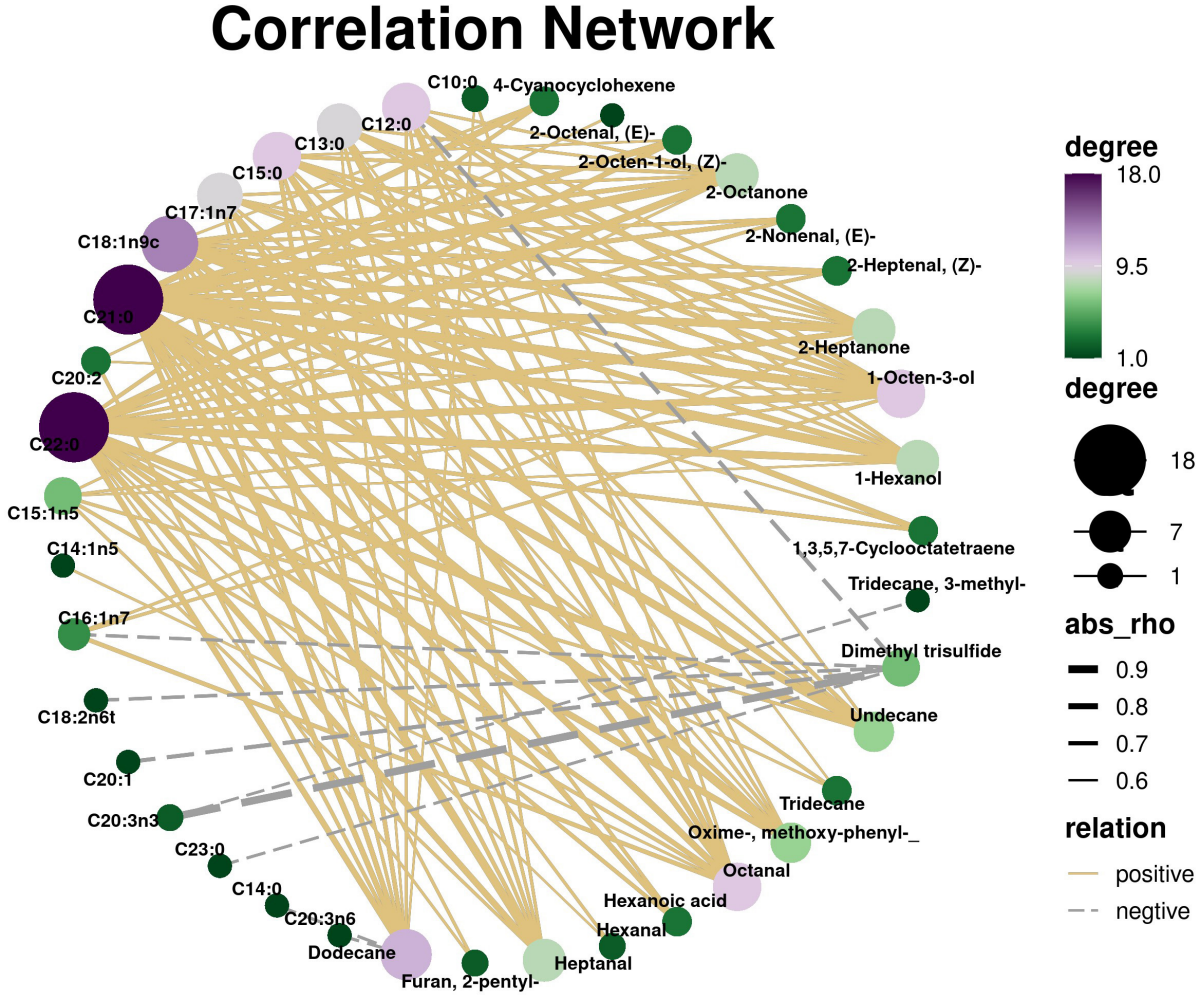
Correlation network analysis of fatty acids and VOCs in six beef samples (*P* < 0.01). Correlation network constructed using Pearson's correlation coefficient.

To systematically investigate the intrinsic relationship between fatty acid composition and volatile flavor compounds in different cuts of roast beef, Pearson correlation network analysis was employed. As shown in Figure 5, the results reveal extensive significant positive correlations (*P* < 0.01) between fatty acids and volatile compounds. The correlation network analysis revealed significant positive correlations between the ultra-long-chain saturated fatty acids (≥C20) C21:0 and C22:0 and most key ketones (2-octanone, 2-heptanone), alcohols (1-octen-3-ol, 1-hexanol), and aldehydes (heptanal, octanal) (*P* < 0.001). These compounds further transform into key flavor compounds contributing fruity, grassy, and mushroom aromas, collectively forming the fat and fruity aroma base of roast beef. Concurrently, medium-chain saturated fatty acids (C12:0, C15:0) and the primary monounsaturated fatty acid C18:1n9c (oleic acid) formed a dense positive correlation network. The specific cleavage of oleic acid hydroperoxide at high temperatures directly yields compounds such as 1-octen-3-ol characteristic of mushroom aroma and 2-heptanone contributing to fat aroma.

Compared to the extensive positive correlations, negative correlations exhibited high specificity (*P* < 0.01), primarily concentrated in significant negative correlations between dimethyl trisulfide and polyunsaturated fatty acids such as C20:3n3 and C18:2n6t. This effectively explains why the risk of cabbage-like off-flavors is reduced in systems with higher polyunsaturated fatty acid content (18). This finding indicates that lipid matrices not only directly participate in flavor formation but also indirectly influence the overall aroma profile by regulating other flavor-generating pathways.

## 4 Discussion

### 4.1 Role of fatty acid composition in roast flavor formation across distinct beef cuts

#### 4.1.1 Validation of the classical fatty acid oxidation pathway and beef cut variability

This study conducted fatty acidomics and VOCs correlation analysis across six distinct beef cuts. Our findings align strongly with established fatty acid oxidation mechanisms. For instance, the SR cut of MUFA produced the highest levels of 1-octen-3-ol (mushroom flavor) and multiple aldehydes. This aligns with the classical mechanism where oleic acid (C18:1n9c) generates 1-octen-3-ol via the 10-hydroperoxide pathway (19). Similarly, the higher linoleic acid (C18:2n6c) content in CR showed a significant correlation with its characteristic hexanal (fresh, green flavor) and 2-pentylfuran (bean, nutty aroma), originating from the cleavage of linoleic acid 13-hydroperoxide (20, 21). These findings confirm that inherent fatty acid composition differences among distinct beef cuts fundamentally shape the positive flavor fingerprints upon heating (22).

#### 4.1.2 Correlation analysis reveals novel associations

The core novel finding of this study lies in uncovering a significant positive correlation (*P* < 0.01) between ultra-long-chain saturated fatty acids (ULCFAs, ≥ C20) which have received limited attention in traditional research and key flavor ketones. Correlation network analysis indicates that C21:0 and C22:0, which are significantly abundant in SR, are highly correlated with 2-octanone (fruity, cheesy) and 2-heptanone (fruity, cheesy). This association has been less systematically reported in previous studies focusing on UFA oxidation (23–25). The potential mechanism may involve the thermal oxidation of these saturated fatty acids (26). Under high-temperature baking conditions, lipid oxidation generates alkoxy radicals via homolysis of hydroperoxides, which undergo β-scission to form methyl ketones (27), which expands our understanding of the range of flavor precursors in beef.

#### 4.1.3 The dual role and competitive mechanism of PUFAs

Another significant finding of this study is the revelation of the dual role PUFAs may play in flavor formation. On one hand, PUFAs (such as C20:3n3, C18:2n6t) serve as precursors for oxidized flavor compounds like aldehydes. On the other hand, our correlation network analysis revealed that these PUFAs exhibit a significant negative correlation with the undesirable sulfur-containing flavor compound dimethyltrisulfide (cabbage flavor) (28). This supports a competitive inhibition hypothesis: during heating, highly reactive PUFAs undergo rapid oxidation preferentially, not only consuming oxygen and free radicals in the system but also generating reactive carbonyl compounds ((E,E)-2,4-decadienal) that sequester hydrogen sulfide (H_2_S) produced from the degradation of sulfur-containing amino acids. This precursor trapping effect redirects H_2_S toward pathways forming other heterocyclic compounds, such as lipid-Maillard interactions, thereby competitively inhibiting the formation of sulfide off-flavor compounds like dimethyl trisulfide (29, 30). This provides a novel chemical perspective explaining why certain PUFA-rich regions (e.g., CR) exhibit lower off-flavor risks.

### 4.2 Comparison and contributions to flavor formation mechanisms

**Figure 6:**
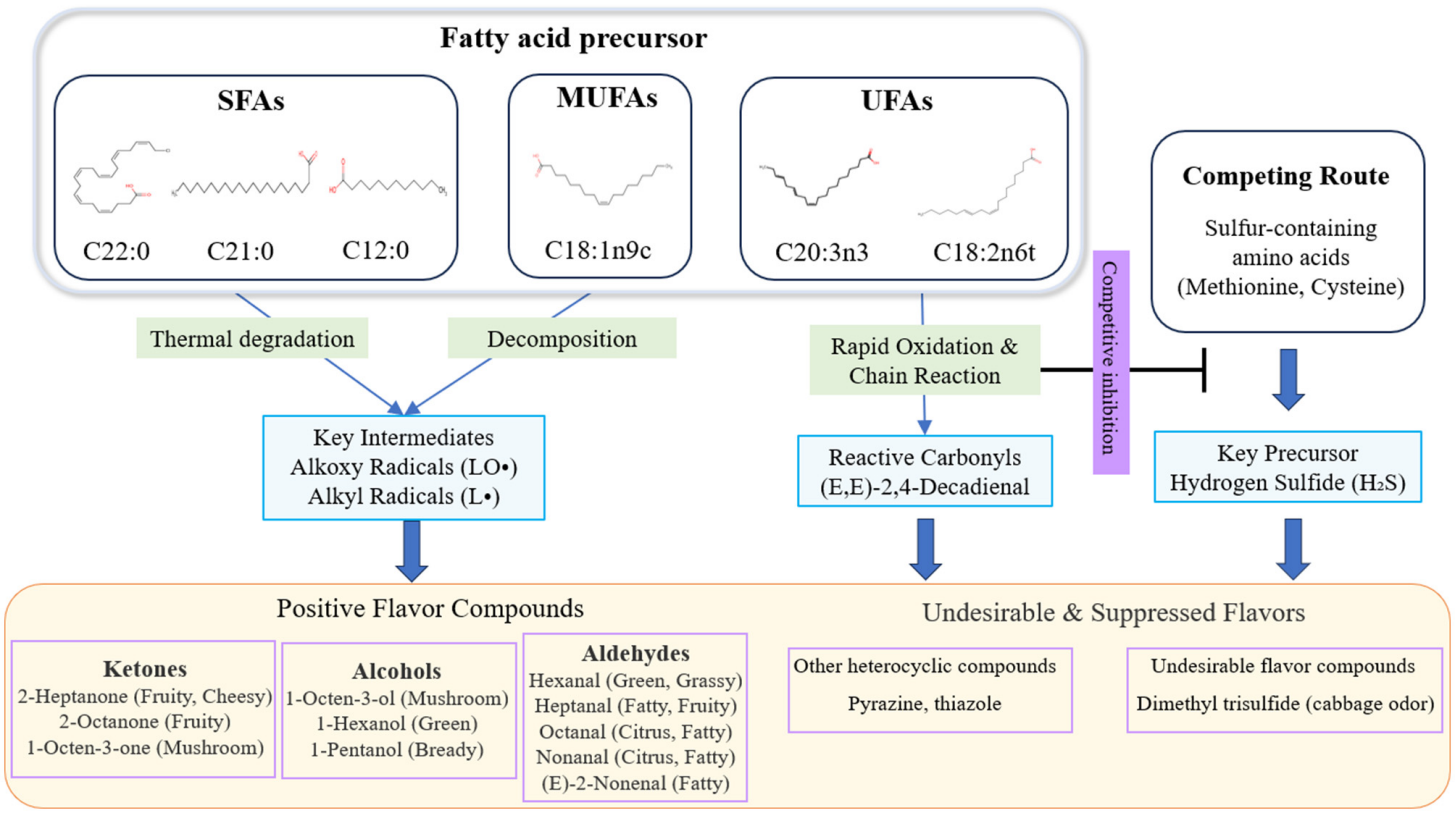
Formation pathway associating fatty acids and VOCs in roasted beef. SFAs, Saturated fatty acids; MUFAs, Monounsaturated fatty acids; PUFAs, Polyunsaturated fatty acids.

Compared to previous studies focused on the oxidation pathways of UFAs, this research expands our understanding of beef roasting flavor formation mechanisms across multiple dimensions. As shown in Figure 6, first, regarding key precursor substances, existing consensus generally recognizes UFAs such as oleic acid (C18:1n9c) and linoleic acid (C18:2n6c) as core flavor precursors (13). This study, for the first time, systematically identifies ultra-long-chain saturated fatty acids (C21:0, C22:0) previously less studied as potential key precursors strongly correlated with characteristic ketone flavors like 2-octanone through a correlation network as potential key precursors strongly correlated with characteristic ketone flavors like 2-octanone, thereby broadening the scope of recognized key flavor precursors (31). Second, regarding the role of PUFA, classical literature predominantly emphasizes their association with undesirable flavors like rancidity through oxidation., However, this study proposes a novel perspective based on significant negative correlations: PUFAs may indirectly inhibit the formation of sulfur-containing off-flavors like dimethyl trisulfide by competitively consuming reaction intermediates within the complex beef matrix, revealing their positive and intricate role in flavor regulation (32, 33). Furthermore, while extensive mechanistic evidence originates from simplified pure lipid model systems, this study's comparative analysis of six authentic commercial beef cuts brings conclusions closer to real-world food systems. Ultimately, these findings collectively point toward a novel bidirectional flavor regulation strategy: not only can specific SFA/MUFA enrichment enhance positive flavors, but PUFA-mediated competitive inhibition can also actively mitigate potential off-flavors. This provides a more comprehensive theoretical basis for the precise customization of beef flavor (34).

### 4.3 Application prospects, limitations, and future directions

The robust correlation map of cut-fatty acid-volatile compounds established in this study provides direct theoretical support and potential targets for quality control and targeted customization of beef flavor. For instance, by selecting raw materials rich in C18:1n9c and C21:0/C22:0, beef products with pronounced mushroom, cheese, and fruity flavors can be purposefully produced (35, 36). Furthermore, strategically incorporating specific plant oils to adjust fatty acid composition in formulations may enable flavor editing (Table 4) (37, 38).

**Table 4:** Strategies for directionally enhancing the flavor of roasted beef via fatty acid addition.

**Target aroma**	**Key target VOCs**	**Recommended fatty acids to add**	**Mechanism and evidence**	**Potential oil sources**
Fruity, cheesy	2-Octanone, 2-Heptanone	Heneicosanoic acid (C21:0), Behenic acid (C22:0)	Thermal degradation and cleavage of very-long-chain SFAs. Directly evidenced by SR tissue data.	Peanut oil (43), rice bran oil (44)
Mushroom, earthy	1-Octen-3-ol	Oleic acid (C18:1n9c)	Major precursor via 10-hydroperoxide. Strong correlation in SR and SS tissues.	High-oleic sunflower oil (45), olive oil (46)
Green, grassy	Hexanal	Linoleic acid (C18:2n6c)	Primary precursor via 13-hydroperoxide cleavage. Directly evidenced by CR tissue data.	Soybean oil, corn oil, sunflower oil (47)
Earthy, nutty	2-Pentylfuran	Linoleic acid (C18:2n6c)	Characteristic oxidation product of linoleic acid. Confirmed by its presence in the CR tissue.	Soybean oil, corn oil, sunflower oil (47)
Fatty, citrusy	Octanal, Nonanal	Oleic acid (C18:1n9c)	Important precursors for these aldehydes. Supported by data from SR and OB tissues.	High-oleic sunflower oil (45), olive oil (46)

Of course, this study has certain limitations and points to directions for future research. First, correlation analysis cannot be equated with causation. Although we have proposed plausible mechanistic hypotheses based on chemical principles, the precise thermochemical pathways by which ultra-long-chain saturated fatty acids generate specific ketones, as well as the quantitative contribution of PUFA competitive inhibition, still require direct validation through model system experiments (e.g., using isotopically labeled fatty acids) (39). Second, our volatile compound data are semi-quantitative. Within complex meat matrices, the release and perception of flavor compounds are influenced by protein-fat interactions, potentially leading to overestimation of calculated aroma activity values. Future work should incorporate sensory evaluation for calibration (40). Finally, sample size remains a constraint in exploratory studies, confirmatory research requires increased biological replicates to enhance statistical validity (41).

Nevertheless, this work integrates fatty acidomics with flavor chemistry to deepen our molecular-level understanding of beef cut-specific flavor formation (42). It establishes a robust scientific foundation for optimizing beef product flavors through precision nutrition strategies or processing additives.

## 5 Conclusion

This study systematically analyzed the relationship between fatty acid composition and volatile flavor compounds during the grilling process of beef from six distinct cuts, revealing the pivotal role of lipid oxidation in aroma formation. Results indicate that fatty acid profiles of different cuts directly influence the aroma spectrum of grilled beef. Through correlation network analysis and multivariate statistical methods, this research identified 23 fatty acids, C18:1n9c, C21:0, and C22:0 with the formation of 30 key aroma compounds, including 1-octen-3-ol, 2-octanone, and 2-heptanone. During roasting, stable SFAs and MUFAs directly contribute positive flavors like fruity, cheesy, and mushroom aromas via thermal degradation pathways; while highly reactive PUFAs competitively inhibit the formation of undesirable sulfur compounds like dimethyl trisulfide through preferential oxidation, thereby bidirectionally regulating the overall aroma profile of roasted beef. This study provides theoretical guidance and practical evidence for achieving targeted flavor quality control in roasted beef through adjustments in fatty acid composition.

## Data Availability

The original contributions presented in the study are included in the article/Supplementary material, further inquiries can be directed to the corresponding author.
