# Supplementary material for: Correlations between fatty acids and key aroma compounds in roasted beef cuts for flavor customization
Source: Front Nutr. 2026 Feb 2;13:1732709. doi: 10.3389/fnut.2026.1732709 (PMC12908179; doi:10.3389/fnut.2026.1732709)
Supplement: Supplementary file 1 [file Table_1.docx]

**Supporting materials for**

**Correlations between Fatty Acids and Key Aroma Compounds in Roasted Beef Cuts for Flavor Customization**

Ningbo Wang^1,2,3*^, Yingying Zhong^2^, Haiqiang Zhu^3^ and Haiying Zhao^3^

^1^ *School of Food Science and Biotechnology,* *Zhejiang Gongshang University, Hangzhou 310018, China*

^2^ *Ningbo Customs Technology Center, Ningbo 315012, China*

^3^ *Ningbo Joysun Product Testing Service Company, Ningbo 315012, China*

*Corresponding Author: Prof. Ningbo Wang

Zhejiang Gongshang University

No.18, Xuezheng Road, Qiantang District, Hangzhou, China, 310018

E-mail: wingning@163.com

Phone: +86-0574-87022701

Table S1. Moisture content and fat content in six beef tissues (means ± SD, n = 3).

| **Beef Cut** | **Moisture Content (%)** | **Crude Fat Content (%)** |
| --- | --- | --- |
| **Chuck Roll (CR)** | 68.2 ± 1.5 | 10.5 ± 1.2 |
| **Short Ribs (SR)** | 59.0 ± 2.1 | 15.5 ± 2.5 |
| **Skirt Steak (SS)** | 64.8 ± 1.8 | 13.3 ± 1.5 |
| **Top Round (TR)** | 73.5 ± 1.2 | 4.2 ± 0.7 |
| **Ribeye Cap (RC)** | 62.5 ± 1.9 | 14.1 ± 1.8 |
| **Oyster Blade (OB)** | 66.0 ± 1.6 | 9.8 ± 1.3 |

| **Name (Abbreviation)** | **CAS number** | **Calibration curves** | **R²** | **LOD (μg/mL)** |
| --- | --- | --- | --- | --- |
| Butyric acid (C4:0) | 623-42-7 | y = 99.83x + 2.14 | 0.998 | 0.185 |
| Caproic acid (C6:0) | 106-70-7 | y = 100.12x - 1.86 | 0.997 | 0.161 |
| Caprylic acid (C8:0) | 111-11-5 | y = 98.76x + 4.23 | 0.995 | 0.161 |
| Capric acid (C10:0) | 110-42-9 | y = 101.05x - 3.17 | 0.999 | 0.162 |
| Undecanoic acid (C11:0) | 1731-86-8 | y = 99.34x + 1.89 | 0.996 | 0.165 |
| Lauric acid (C12:0) | 111-82-0 | y = 100.67x - 2.45 | 0.998 | 0.157 |
| Tridecylic acid (C13:0) | 1731-88-0 | y = 98.92x + 3.56 | 0.994 | 0.158 |
| Myristic acid (C14:0) | 124-10-7 | y = 99.78x + 2.01 | 0.997 | 0.129 |
| Myristoleic acid (C14:1n5) | 56219-06-8 | y = 101.23x - 4.12 | 0.999 | 0.162 |
| Pentadecanoic acid (C15:0) | 7132-64-1 | y = 98.45x + 5.34 | 0.992 | 0.155 |
| Pentadecenoic acid (C15:1n5) | 90176-52-6 | y = 100.34x - 1.23 | 0.998 | 0.159 |
| Palmitic acid (C16:0) | 112-39-0 | y = 99.12x + 3.78 | 0.995 | 0.171 |
| Palmitoleic acid (C16:1n7) | 1120-25-8 | y = 100.89x - 3.45 | 0.998 | 0.157 |
| Margaric acid (C17:0) | 1731-92-6 | y = 98.67x + 4.89 | 0.993 | 0.148 |
| Heptadecenoic acid (C17:1n7) | 75190-82-8 | y = 101.17x - 4.56 | 0.999 | 0.155 |
| Stearic acid (C18:0) | 112-61-8 | y = 99.45x + 2.34 | 0.996 | 0.169 |
| Elaidic acid (C18:1n9t) | 1937-62-8 | y = 100.23x - 1.67 | 0.997 | 0.152 |
| Oleic acid (C18:1n9c) | 112-62-9 | y = 98.89x + 4.12 | 0.994 | 0.165 |
| Linolelaidic acid (C18:2n6t) | 2566-97-4 | y = 100.56x - 2.89 | 0.998 | 0.153 |
| Linoleic acid (C18:2n6c) | 112-63-0 | y = 99.67x + 2.45 | 0.996 | 0.135 |
| Arachidic acid (C20:0) | 1120-28-1 | y = 98.34x + 6.23 | 0.991 | 0.15 |
| γ-Linolenic acid (C18:3n6) | 16326-32-2 | y = 100.78x - 3.12 | 0.998 | 0.156 |
| Gadoleic acid (C20:1) | 2390-09-2 | y = 99.23x + 3.34 | 0.995 | 0.156 |
| α-Linolenic acid (C18:3n3) | 301-00-8 | y = 101.34x - 5.23 | 0.999 | 0.163 |
| Heneicosanoic acid (C21:0) | 6064-90-0 | y = 99.89x + 2.12 | 0.997 | 0.157 |
| Eicosadienoic acid (C20:2) | 61012-46-2 | y = 98.56x + 5.67 | 0.992 | 0.172 |
| Behenic acid (C22:0) | 929-77-1 | y = 100.45x - 2.34 | 0.998 | 0.143 |
| Dihomo-γ-linolenic acid (C20:3n6) | 21061-10-9 | y = 99.78x + 2.89 | 0.996 | 0.182 |
| Erucic acid (C22:1n9) | 1120-34-9 | y = 100.12x - 1.45 | 0.997 | 0.164 |
| Mead acid (C20:3n3) | 55682-88-7 | y = 98.93x + 4.23 | 0.994 | 0.185 |
| Tricosanoic acid (C23:0) | 2566-89-4 | y = 99.45x + 3.12 | 0.996 | 0.13 |
| Arachidonic acid (ARA, C20:4n6) | 2433-97-8 | y = 101.23x - 4.89 | 0.999 | 0.139 |
| Docosadienoic acid (C22:2n6) | 61012-47-3 | y = 99.34x + 2.67 | 0.996 | 0.187 |
| Lignoceric acid (C24:0) | 2442-49-1 | y = 100.56x - 2.12 | 0.998 | 0.195 |
| Eicosapentaenoic acid (EPA, C20:5n3) | 2734-47-6 | y = 98.67x + 5.34 | 0.993 | 0.218 |
| Nervonic acid (C24:1n9) | 2733-88-2 | y = 100.89x - 3.45 | 0.998 | 0.207 |
| Docosahexaenoic acid methyl ester (DHA, C22:6n3) | 2566-90-7 | y = 99.12x + 3.78 | 0.995 | 0.318 |

Table S2. Calibration curves, recoveries, and LOD values for 37 fatty acid determinations.

Table S3. Detailed results of one-way ANOVA with Tukey’s HSD post hoc tests for pairwise comparisons of fatty acid contents among beef cuts. The table lists the statistical comparisons for total fatty acids (FAs), saturated fatty acids (SFAs), monounsaturated fatty acids (MUFAs), and polyunsaturated fatty acids (PUFAs). For each comparison between two cuts, the Mean Difference (Mean Diff.), 95% Confidence Interval (95% CI) of the difference, and the Adjusted P Value (adjusted for multiple comparisons by Tukey’s method).

|  | **Tukey's multiple comparisons test** | **Mean Diff.** | **95.00% CI of diff.** | **Adjusted P Value** |
| --- | --- | --- | --- | --- |
| **FAs** | CR vs. TR | 1006 | 309.8 to 1702 | 0.0041 |
|  | CR vs. SS | -664.9 | -1361 to 31.27 | 0.0643 |
|  | CR vs. SR | -2944 | -3640 to -2248 | <0.0001 |
|  | CR vs. RC | -1063 | -1760 to -367.2 | 0.0026 |
|  | CR vs. OB | 40.9 | -655.3 to 737.1 | >0.9999 |
|  | TR vs. SS | -1671 | -2367 to -974.7 | <0.0001 |
|  | TR vs. SR | -3950 | -4646 to -3254 | <0.0001 |
|  | TR vs. RC | -2069 | -2766 to -1373 | <0.0001 |
|  | TR vs. OB | -965.1 | -1661 to -268.9 | 0.0057 |
|  | SS vs. SR | -2279 | -2975 to -1583 | <0.0001 |
|  | SS vs. RC | -398.4 | -1095 to 297.7 | 0.4346 |
|  | SS vs. OB | 705.8 | 9.630 to 1402 | 0.0463 |
|  | SR vs. RC | 1881 | 1185 to 2577 | <0.0001 |
|  | SR vs. OB | 2985 | 2289 to 3681 | <0.0001 |
|  | RC vs. OB | 1104 | 408.1 to 1800 | 0.0019 |
| **SFAs** | CR vs. TR | 169.9 | 152.8 to 187.1 | <0.0001 |
|  | CR vs. SS | -91.34 | -108.5 to -74.15 | <0.0001 |
|  | CR vs. SR | -295.1 | -312.2 to -277.9 | <0.0001 |
|  | CR vs. RC | 87.35 | 70.16 to 104.5 | <0.0001 |
|  | CR vs. OB | -66.46 | -83.65 to -49.27 | <0.0001 |
|  | TR vs. SS | -261.3 | -278.5 to -244.1 | <0.0001 |
|  | TR vs. SR | -465 | -482.2 to -447.8 | <0.0001 |
|  | TR vs. RC | -82.59 | -99.78 to -65.40 | <0.0001 |
|  | TR vs. OB | -236.4 | -253.6 to -219.2 | <0.0001 |
|  | SS vs. SR | -203.7 | -220.9 to -186.5 | <0.0001 |
|  | SS vs. RC | 178.7 | 161.5 to 195.9 | <0.0001 |
|  | SS vs. OB | 24.88 | 7.692 to 42.07 | 0.004 |
|  | SR vs. RC | 382.4 | 365.2 to 399.6 | <0.0001 |
|  | SR vs. OB | 228.6 | 211.4 to 245.8 | <0.0001 |
|  | RC vs. OB | -153.8 | -171.0 to -136.6 | <0.0001 |
| **MUFAs** | CR vs. TR | 276.1 | -363.8 to 915.9 | 0.6994 |
|  | CR vs. SS | -1109 | -1749 to -469.6 | 0.0009 |
|  | CR vs. SR | -2995 | -3635 to -2355 | <0.0001 |
|  | CR vs. RC | -1703 | -2342 to -1063 | <0.0001 |
|  | CR vs. OB | -403.5 | -1043 to 236.3 | 0.34 |
|  | TR vs. SS | -1385 | -2025 to -745.6 | 0.0001 |
|  | TR vs. SR | -3271 | -3911 to -2631 | <0.0001 |
|  | TR vs. RC | -1979 | -2618 to -1339 | <0.0001 |
|  | TR vs. OB | -679.6 | -1319 to -39.75 | 0.0352 |
|  | SS vs. SR | -1886 | -2526 to -1246 | <0.0001 |
|  | SS vs. RC | -593.1 | -1233 to 46.68 | 0.0751 |
|  | SS vs. OB | 705.9 | 66.07 to 1346 | 0.0279 |
|  | SR vs. RC | 1293 | 652.8 to 1932 | 0.0002 |
|  | SR vs. OB | 2592 | 1952 to 3231 | <0.0001 |
|  | RC vs. OB | 1299 | 659.2 to 1939 | 0.0002 |
| **PUFAs** | CR vs. TR | 563.3 | 547.0 to 579.6 | <0.0001 |
|  | CR vs. SS | 482.8 | 466.5 to 499.1 | <0.0001 |
|  | CR vs. SR | 301.7 | 285.4 to 318.0 | <0.0001 |
|  | CR vs. RC | 552.9 | 536.6 to 569.1 | <0.0001 |
|  | CR vs. OB | 521.1 | 504.8 to 537.3 | <0.0001 |
|  | TR vs. SS | -80.48 | -96.77 to -64.19 | <0.0001 |
|  | TR vs. SR | -261.6 | -277.9 to -245.3 | <0.0001 |
|  | TR vs. RC | -10.46 | -26.75 to 5.830 | 0.3231 |
|  | TR vs. OB | -42.26 | -58.55 to -25.97 | <0.0001 |
|  | SS vs. SR | -181.1 | -197.4 to -164.8 | <0.0001 |
|  | SS vs. RC | 70.02 | 53.73 to 86.31 | <0.0001 |
|  | SS vs. OB | 38.22 | 21.93 to 54.51 | <0.0001 |
|  | SR vs. RC | 251.1 | 234.8 to 267.4 | <0.0001 |
|  | SR vs. OB | 219.3 | 203.0 to 235.6 | <0.0001 |
|  | RC vs. OB | -31.8 | -48.09 to -15.51 | 0.0003 |

Table S4. VIP value list for the OPLS-DA analysis model of fatty acids in six beef tissue tissues.

| **Var ID (Primary)** | **VIP** |
| --- | --- |
| C20:3n3 | 1.21297 |
| C18:2n6t | 1.13572 |
| C18:2n6c | 1.13103 |
| C18:3n3 | 1.1043 |
| C15:1n5 | 1.0956 |
| C23:0 | 1.09509 |
| C17:0 | 1.09137 |
| C18:1n9c | 1.06107 |
| C20:0 | 1.04725 |
| C13:0 | 1.01297 |
| C21:0 | 1.00224 |
| C14:0 | 0.976601 |
| C14:1n5 | 0.975116 |
| C18:3 n3 | 0.969842 |
| C20:3n6 | 0.968758 |
| C22:0 | 0.966017 |
| C22:1n9 | 0.950541 |
| C16:1n7 | 0.914963 |
| C20:1 | 0.892718 |
| C20:2 | 0.886411 |
| C17:1n7 | 0.86892 |
| C12:0 | 0.846347 |
| C10:0 | 0.837117 |
| C15:0 | 0.827023 |

Table S5. Detailed results of one-way ANOVA with Tukey’s HSD post hoc tests for pairwise comparisons of VOCs contents among beef cuts. For each comparison between two cuts, the Mean Difference (Mean Diff.), 95% Confidence Interval (95% CI) of the difference, and the Adjusted P Value (adjusted for multiple comparisons by Tukey’s method).

| Tukey's multiple comparisons test | Mean Diff. | 95.00% CI of diff. | Adjusted P Value |
| --- | --- | --- | --- |
| CR vs. TR | 0.044 | -0.9188 to 1.007 | >0.9999 |
| CR vs. SS | -1.731 | -2.694 to -0.7685 | 0.0006 |
| CR vs. SR | -5.716 | -6.679 to -4.753 | <0.0001 |
| CR vs. RC | 0.03967 | -0.9231 to 1.002 | >0.9999 |
| CR vs. OB | -0.2683 | -1.231 to 0.6945 | 0.9292 |
| TR vs. SS | -1.775 | -2.738 to -0.8125 | 0.0005 |
| TR vs. SR | -5.76 | -6.723 to -4.797 | <0.0001 |
| TR vs. RC | -0.004333 | -0.9671 to 0.9585 | >0.9999 |
| TR vs. OB | -0.3123 | -1.275 to 0.6505 | 0.8764 |
| SS vs. SR | -3.985 | -4.947 to -3.022 | <0.0001 |
| SS vs. RC | 1.771 | 0.8082 to 2.734 | 0.0005 |
| SS vs. OB | 1.463 | 0.5002 to 2.426 | 0.0027 |
| SR vs. RC | 5.756 | 4.793 to 6.718 | <0.0001 |
| SR vs. OB | 5.448 | 4.485 to 6.410 | <0.0001 |
| RC vs. OB | -0.308 | -1.271 to 0.6548 | 0.8824 |

Table S6. VIP value list obtained from the OPLS-DA analysis model for VOCs.

| **Var ID (Primary)** | **VIP** |
| --- | --- |
| 2,3-Octanedione | 1.32686 |
| Octadecane, 2-methyl- | 1.3124 |
| Undecane, 3-methyl- | 1.26669 |
| Nonadecane, 2-methyl- | 1.23372 |
| Dimethyl trisulfide | 1.17022 |
| Benzaldehyde | 1.16195 |
| Phenol, 2,4-bis(1,1-dimethylethyl)- | 1.12007 |
| Hexadecane | 1.10872 |
| Propane, 2,2'-[methylenebis(oxy)]bis[2-methyl- | 1.08034 |
| Dodecane | 1.07983 |
| Tridecane, 3-methyl- | 1.04784 |
| 4-Cyanocyclohexene | 0.996326 |
| Undecane | 0.984767 |
| 1,3,5,7-Cyclooctatetraene | 0.970791 |
| Octanal | 0.968574 |
| 1-Octen-3-ol | 0.930257 |
| 2-Octanone | 0.928895 |
| Oxime-, methoxy-phenyl-_ | 0.922949 |
| 1-Hexanol | 0.912896 |
| Heptanal | 0.909562 |
| 2-Heptanone | 0.903597 |
| Nonanal | 0.895964 |
| Tridecane | 0.891738 |
| 2-Octen-1-ol, (Z)- | 0.864242 |
| 2-Octenal, (E)- | 0.847929 |
| 2-Nonenal, (E)- | 0.846773 |
| 2-Heptenal, (Z)- | 0.842131 |
| Hexanal | 0.83552 |
| Tetradecane | 0.789426 |
| Furan, 2-pentyl- | 0.736984 |
| 2-Butanone, 3,4-epoxy-3-ethyl- | 0.708003 |


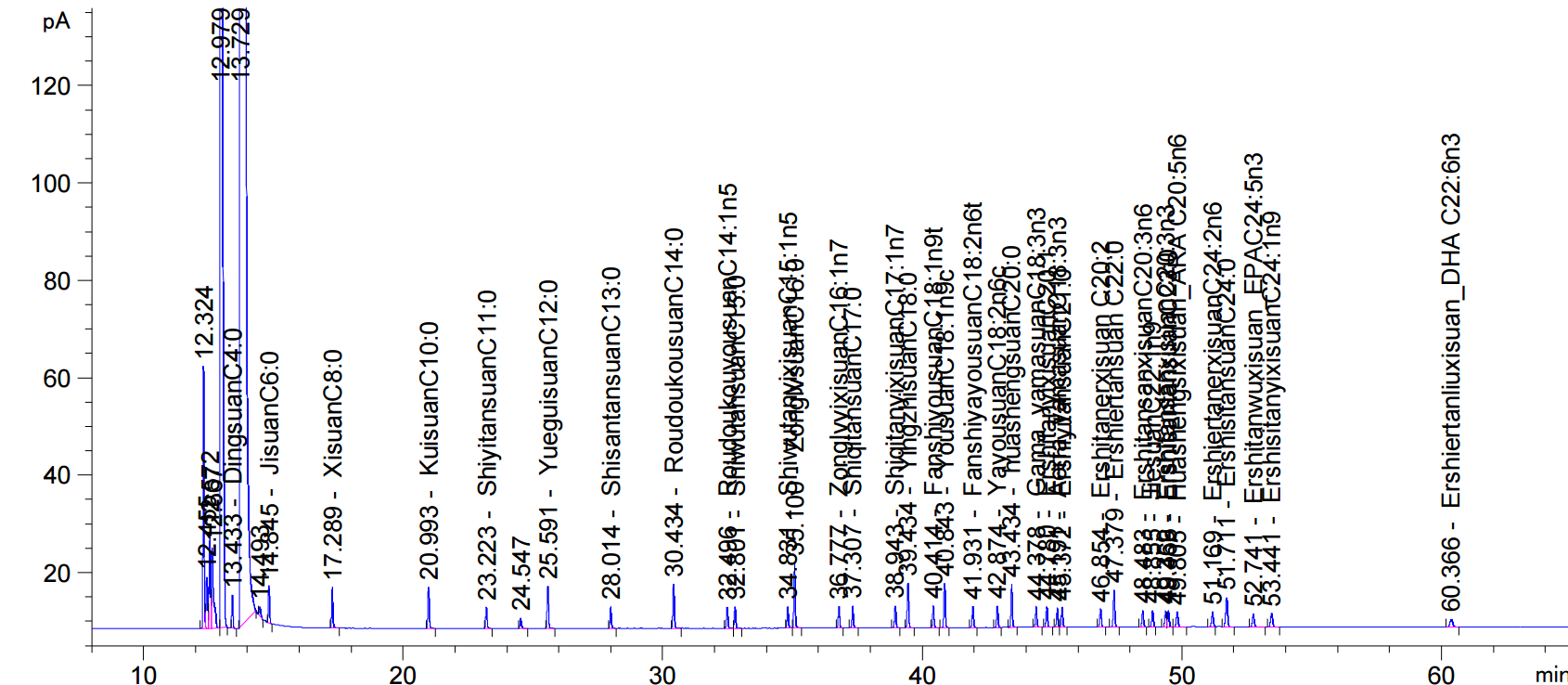


Figure S1. Chromatogram of 37 fatty acid standards.


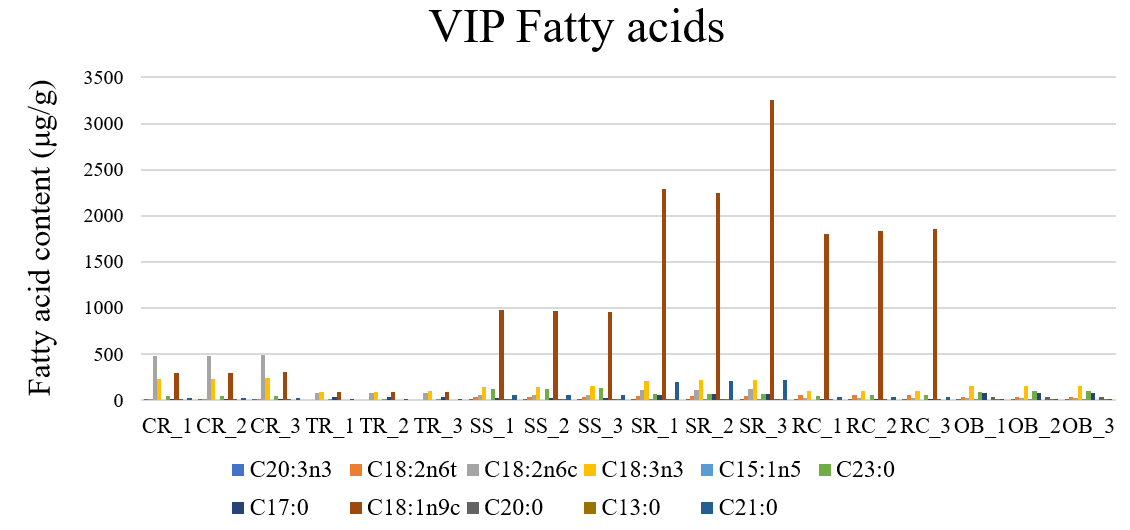


Figure S2. VIP fatty acids in six types of roast beef tissue.


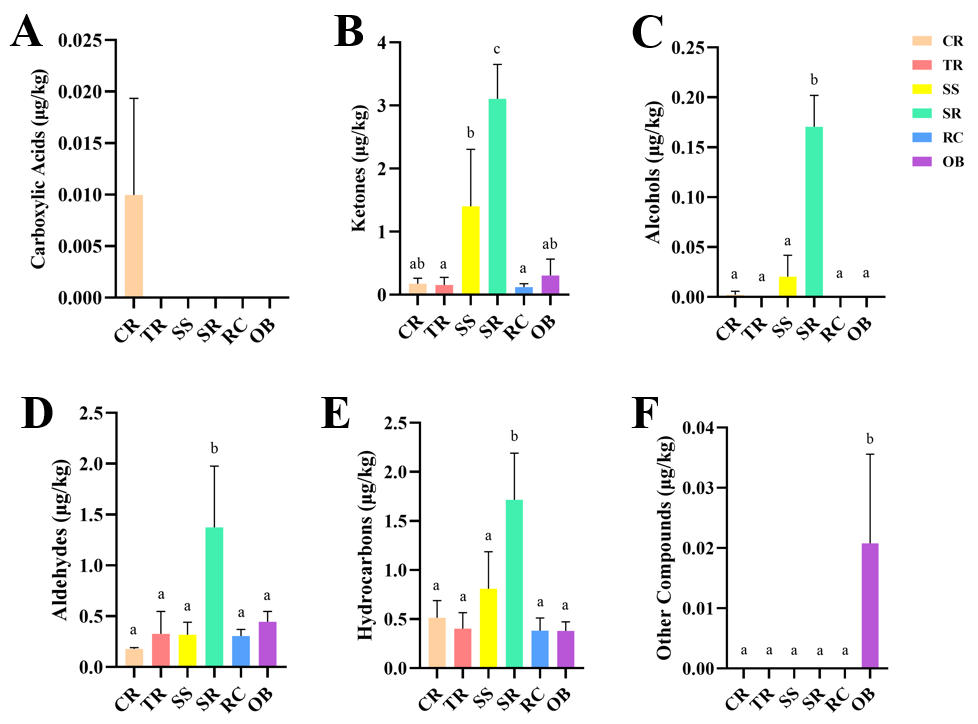


Figure S3. Analysis of Specific Compound Categories of Volatile Organic Compounds (means ± SD, n = 3). Significant differences (P < 0.05; one-way ANOVA, Tukey’s HSD test) among cuts within indicated by different lowercase letters.
